# Calcium Imaging of AM Dyes Following Prolonged Incubation in Acute Neuronal Tissue

**DOI:** 10.1371/journal.pone.0155468

**Published:** 2016-05-16

**Authors:** Morven Cameron, Orsolya Kékesi, John W. Morley, Jonathan Tapson, Paul P. Breen, André van Schaik, Yossi Buskila

**Affiliations:** 1 School of Medicine, Western Sydney University, Penrith, NSW, Australia; 2 Biomedical Engineering and Neuroscience group, The MARCS Institute, Western Sydney University, Penrith, NSW, Australia; Cinvestav-IPN, MEXICO

## Abstract

Calcium-imaging is a sensitive method for monitoring calcium dynamics during neuronal activity. As intracellular calcium concentration is correlated to physiological and pathophysiological activity of neurons, calcium imaging with fluorescent indicators is one of the most commonly used techniques in neuroscience today. Current methodologies for loading calcium dyes into the tissue require prolonged incubation time (45–150 min), in addition to dissection and recovery time after the slicing procedure. This prolonged incubation curtails experimental time, as tissue is typically maintained for 6–8 hours after slicing. Using a recently introduced recovery chamber that extends the viability of acute brain slices to more than 24 hours, we tested the effectiveness of calcium AM staining following long incubation periods post cell loading and its impact on the functional properties of calcium signals in acute brain slices and wholemount retinae. We show that calcium dyes remain within cells and are fully functional >24 hours after loading. Moreover, the calcium dynamics recorded >24 hrs were similar to the calcium signals recorded in fresh tissue that was incubated for <4 hrs. These results indicate that long exposure of calcium AM dyes to the intracellular cytoplasm did not alter the intracellular calcium concentration, the functional range of the dye or viability of the neurons. This data extends our previous work showing that a custom recovery chamber can extend the viability of neuronal tissue, and reliable data for both electrophysiology and imaging can be obtained >24hrs after dissection. These methods will not only extend experimental time for those using acute neuronal tissue, but also may reduce the number of animals required to complete experimental goals.

## Introduction

### Calcium signalling in the central nervous system

Calcium is one of the most common second messengers in the brain, playing a key role in a variety of intracellular physiological processes, ranging from cell proliferation [1] to synaptic plasticity [2] and cell death [3]. At rest, the intracellular calcium concentration in the cytoplasm is ~50–100 nM and can rise quickly following stimulation (e.g. electrical activity or agonist) to levels that are ten times higher [4]. An intracellular increase in free calcium concentration modulates the activity of numerous proteins, including kinases, phosphatases, transcription factors and enzymes, which participate in many physiological and pathological processes (reviewed by [4]). Due to calcium importance in these processes, calcium imaging is widely used in many laboratories over the world, with more than half a million manuscripts published over the last few decades.

Calcium signals occur within cells (intracellular, as above) and also between cells [5]. Intercellular calcium signalling takes place in two ways: direct transmission through gap junctions–mainly in glial cells [6], or through transmitter gated channels (e.g. NMDA and voltage gated calcium channels)[5], in which neurotransmitter release from a presynaptic cell leads to a cascade of processes in the postsynaptic region, that ultimately increase the cytoplasmic calcium concentration. The source of calcium entry to the cytoplasm can be either external (from the extracellular space) or internal (from calcium stores in the endoplasmatic reticulum (ER)). Calcium concentration is tightly regulated by calcium transporters that remove calcium ions rapidly, both avoiding cytotoxic effects [7] and maintaining the 20,000 fold gradient in concentration across the cell membrane [5]. In glial cells, it has been shown that calcium signals fall into two main categories: calcium oscillations, and calcium waves, in which the calcium signal spreads via gap junctions from one astrocyte to another as an inter-glial communication signal [6].

### Calcium imaging in neuronal tissue

Cytosolic Ca^2+^ signals can be classified as ***transient*** (small and brief elevations due to calcium influx through membrane calcium channels), ***sustained*** (high and sustained increase in calcium signal following calcium influx from both external and internal stores) or ***oscillatory signals*** (repeatable brief increase in free calcium in the intracellular cytoplasm)[7]. Functional imaging of calcium as a measure of neuronal activity is a key technique in neuroscience research, and the use of fluorescent dyes is the most efficient and popular tool to study intracellular calcium dynamics in individual neurons and glia. Moreover, it permits investigation of the network activity within the central nervous system (CNS), as it provides good spatial and temporal resolution [8]. Due to the importance of calcium indicators to this technique, they are constantly being improved to increase signal detection through various means such as wider wavelength options, higher fluorescence intensities, less bleaching and lower dissociation constants (K_d_)[4,8,9]. These advances allow faster and more accurate measurements of calcium influx into neurons, which also provides an indirect, but accurate measure of spiking activity [10].

One of the limitations of calcium imaging lies within the calcium indicators, which are unable to cross the lipid membrane easily due to their charged nature. However calcium acetoxymethyl (AM) indicators, which were first introduced by Roger Tsien in the early 80’s [11] can enter the cell through the plasma membrane as the indicators negative charge is masked. Following hydrolysis of the acetyl groups by intracellular esterases, the indicator molecules are released and stay inside the cell, as they cannot pass the membrane in their new charged conformation. Therefore, the efficiency of AM dye loading varies between cells. This can be very challenging in thick tissues, as it requires long loading periods to reach an adequate concentration of dye molecules inside the cells. Hence, during *in-vitro* dye loading of brain slices, the incubation time of the dye is correlated to the age of the animal [12], occasionally requiring >3 hrs for tissue prepared from “aged brain”. Given the amount of time and effort required to develop and raise animals for research, increasing the time available for investigation of the physiological processes in the CNS will be of great benefit.

Acute brain slices conserve various properties of *in vivo* biology, including functional local intercellular circuitry with preserved brain architecture that allows easy access to the studied cells as well as precise control of the extracellular environment. Moreover, slices from the CNS allow direct visualization of the cellular circuit, which enables researchers to locate, identify and easily access the cells being studied. Additionally, it is possible to locally load cells with the dye, which would otherwise be blocked by the blood brain barrier. Calcium imaging in acute brain slices offers a high degree of temporal and spatial resolution [13] and therefore is commonly used to investigate physiological processes. Together with recently developed optical and computational technologies, it allows recording of the network activity and analysis of integrated cellular function [14].

Like brain slices, retinal explants conserve whole tissue networks and are by far the most accessible model for CNS function. However calcium-imaging in the retina has never been a commonly used technique for two main reasons: the exquisite sensitivity of this tissue to visible light, and the difficulty loading retinal neurons with calcium dyes. With new optical breakthroughs such as two-photon microscopy [15] and the development of new techniques that allows for ubiquitous staining of retinal neurons with calcium dyes [14], calcium-imaging in the retina is set to expand considerably in the coming years. Both acute brain slices and retina explants, however, have a limited lifespan (6–12 hrs) that constrains the time available to study the cellular and network physiological properties. We recently reported a new method to extend the viability of acute brain slices (up to 36 hrs) using a recovery chamber that controls and regulates the external environment of the slices [16,17]. Here we extend this study to test the functionality and viability of slices and retinal explants using calcium-imaging techniques.

We present evidence that calcium dyes loaded into retinal wholemount and brain slices remains in cells for more than 24 hours. Moreover, we show that measuring neurophysiological properties using calcium imaging is possible even 24 hrs after slicing and loading the cells, as no significant alterations were detected either in the total number of cells that were loaded with the dye (loaded cells) or the number of activated cells (evoked cells). This study demonstrates that the functional activity of neurons in acute brain slices can be maintained for long periods (>24 hrs), once the external environment is highly regulated, and that calcium AM dyes remain stably loaded and functional for extended periods. The costs and time spent on preparing such experiments are high, and the use of the Braincubator provides a way to maximise this significant investment, by at least tripling the experimental time. Moreover, animal numbers can be significantly reduced, providing strong ethical reasons for the adoption of such methodology.

## Materials and Methods

### Animals

For this study we used 3–30 weeks old Wister rats, C57BL/6 and C3H/HeJArc (*rd/rd*) mice (all born in Western Sydney University animal facility). All animals were healthy and handled with standard conditions of temperature, humidity, 12hr light/ dark cycle, free access to food and water, and without any intended stress stimuli. All experiments were approved and performed in accordance with the University of Western Sydney Animal Care and Ethics committee and according to the animal use and care guidelines (Animal Research Authority #A9452, #A10396 and #A8967).

### Brain slice and Retinal wholemount preparation

Animals were deeply anesthetized by inhalation of isoflurane (5%), decapitated, and their brains quickly removed and placed into ice-cold physiological solution (artificial cerebrospinal fluid (aCSF)) containing (in mM): 125 NaCl, 2.5 KCl, 1 MgCl_2_, 1.25 NaH_2_PO_4_, 2 CaCl_2_, 25 NaHCO_3_, 25 dextrose and saturated with carbogen (95%O_2_−5%CO_2_ mixture; 310 mOsm; pH7.4; [18]). ***Parasagittal brain slices*** (300 μm thick) were cut with a vibrating microtome (Leica VT1200S) as previously described [19,20] and transferred to a custom built incubation system that closely monitored and controlled pH levels, carbogen flow and temperature as well as irradiating bacteria through a separate UV chamber (Braincubator)[17]. Initially the temperature was set to 35°C for 30 min, and then slowly reduced to 16°C until slices were used for experiments.

Retinal wholemounts were prepared under normal laboratory lighting conditions as previously described [21]. Briefly, animals were euthanized using cervical dislocation, eyes enucleated, cut along the ora serrata, and placed in artificial cerebrospinal fluid (aCSF) containing (mM): 125 NaCl, 25 NaHCO_3_, 3 KCl, 2 CaCl_2_, 1 MgCl_2_,10 Glucose and saturated with carbogen (95%O_2_−5%CO_2_ mixture; 300 mOsm; pH7.4; [22]), at room temperature (RT) within 1 min of euthanasia. Retinae were isolated from the surrounding eye tissue, cut to allow the tissue to lie flat, and adhered to 22nm Millipore filter paper. To remove the inner limiting membrane (ILM), a previously published method [23] was modified as follows: the retinae were transferred to solution containing 30U/ml papain, 1mM L-cystine, 0.5mM EDTA and 0.005% DNase in Earl’s balanced salt solution (BSS) (Worthington cat. No. L503126) at 37°C for 20 min and then brought to room temperature slowly rocking for a further 10 min. To stop enzymatic digestion, an ovomucoid (10 mg/ml) and BSA (10 mg/ml) solution was applied for 5 min in Earl’s BSS. Tissue was then washed in aCSF and immediately loaded with calcium dyes before eventual transfer to the Braincubator and maintained at 16°C.

#### Maintaining tissue in the Braincubator

To extend the viability of acute brain slices, we incubated the tissue in a custom-built recovery incubation system, called the Braincubator, as reported previously [16]. This system can monitor and control the external environment of the brain slices (aCSF, as detailed above) through a closed loop circulation system consisting of two chambers. Slices were placed in the main chamber, which contained probes for pH and temperature and was externally covered with aluminium heatproof duct tape for UVC blockade and stop dyes bleaching. The second chamber (UVC chamber) was isolated from the main chamber and exposed to 1.1 W UVC light (254 nm, 5W/2P Philips Ultra Violet sterilizer UV lamp) in order to eradiate bacteria floating in the solution. UVC light timing was controlled via a programmable timer using a random feature that turns ON at times varying between 15 and 26 min every 15 to 30 min. The UVC chamber volume was set to 120 ml, and the flow rate was set to 12 ml/min. Application of UVC was intermittent and randomised to avoid excessive heating of the aCSF temperature and to prevent an increase in bacterial UVC resistance. The UVC chamber was covered with aluminium foil to prevent UVC illumination outside the chamber, which can damage neuronal tissue. A peristaltic pump circulated the solution (aCSF) through the two chambers and a Peltier thermoelectric cold plate cooler (TE technology, Traverse city, MI) enabled the solution to be either cooled or heated to temperatures in the range of 0–50°C.

### Calcium Imaging

#### Calcium dye loading

Calcium dyes (Fura-2 AM or Fluo-4 AM, Life technologies) were dissolved in DMSO (50 μg in 50 μl) and 1% pluronic acid-127 (Molecular Probes), sonicated for 15 min and loaded into the tissue by bath application to a final concentration of 10 μM (brain slices) and 20 μM (retina), as reported by [10]. In retinae and brain slices from young animals, the dye was loaded to the bath for 45 min at 37°C (Fura-2 AM) or at RT (Fluo-4 AM), following protocols reported by [10] and [24] respectively. For adult animals, (>12 weeks) the dyes were loaded directly on the slices, as suggested by [10], and maintained for 75 minutes to allow better penetration of the dye into the deep layers. To ensure adequate oxygenation of the submerged slice during dye incubation, the loading chamber (4 cm diameter; 1ml volume of aCSF) was kept in a closed container that was oxygenated continuously with 95% O_2_-5% CO_2._ Following dye loading, the tissue was washed with aCSF and transferred to the Braincubator until experimental use.

For imaging, tissue was placed in a submerged recording chamber under an Olympus BX51W microscope, and perfused with oxygenated aCSF at a flow rate of 4–5 ml/min at room temperature (~22°C). To prevent the tissue from moving in the fluid stream the slices it was held under a ‘‘harp” (made of nylon or gold threads stretched and glued across a U-shaped piece of gold or platinum wire). Image time series were acquired with water-immersion objectives at different rates ranging from 0.3–3 Hz.

For ratiometric imaging of Fura-2, the excitation light was filtered through an Ultra High Speed Wavelength Switcher (Lambda DG-4 Plus; Sutter instruments, Novato, CA) to provide wavelengths of 340 and 380. Emission light from individual cells was passed through an emission filter of 510 ± 20 nm, and captured by a digital camera (Q-Imaging ROLERA-XR or ANDOR Xion). For a single excitation wavelength of Fluo-4, the excitation light was filtered through a 460–490 nm band pass filter and the emission light passed through a 515–550 nm band pass filter, and captured by a high speed digital camera (NeuroCCD, RedShirtimaging).

Acquisition protocols consisted of 5–7-minute time-lapse sequences of Fura-2 or Fluo-4 fluorescence. Alterations in fluorescence as a function of time were measured at a single wavelength (Fluo-4) or the wavelength ratio method (Fura-2), as previously described by [25]. All analysis and processing, as well as playback of the image sequences for visual inspection, was made using *ImageJ* /FIJI software ((http://fiji.sc/Fiji) [26]. To visualize the spatial and temporal changes in calcium resulting from spontaneous activity, the raw sequences were processed to highlight changes in fluorescence intensity between frames. Regions of interest over the field of view were selected, and the mean pixel intensity at each frame was measured. The data was first plotted as fluorescence intensity versus time (z profile) and subsequently converted to a relative scale (ΔF/F baseline). To allow comparison across slices the same threshold value was used for all slices.

### Statistical analysis

Data is reported as mean ± S.E.M. Analysis of calcium signals was completed using *ImageJ*—FIJI software (NIH). Stained cells were classified as loaded cells, evoked cells or spontaneously active cells according to the staining pattern. An unpaired two-tailed student t-test was used for comparison between different cell populations or different groups of slices. P-values of less than 0.05 were considered statistically significant.

## Results

**Fig 1:**
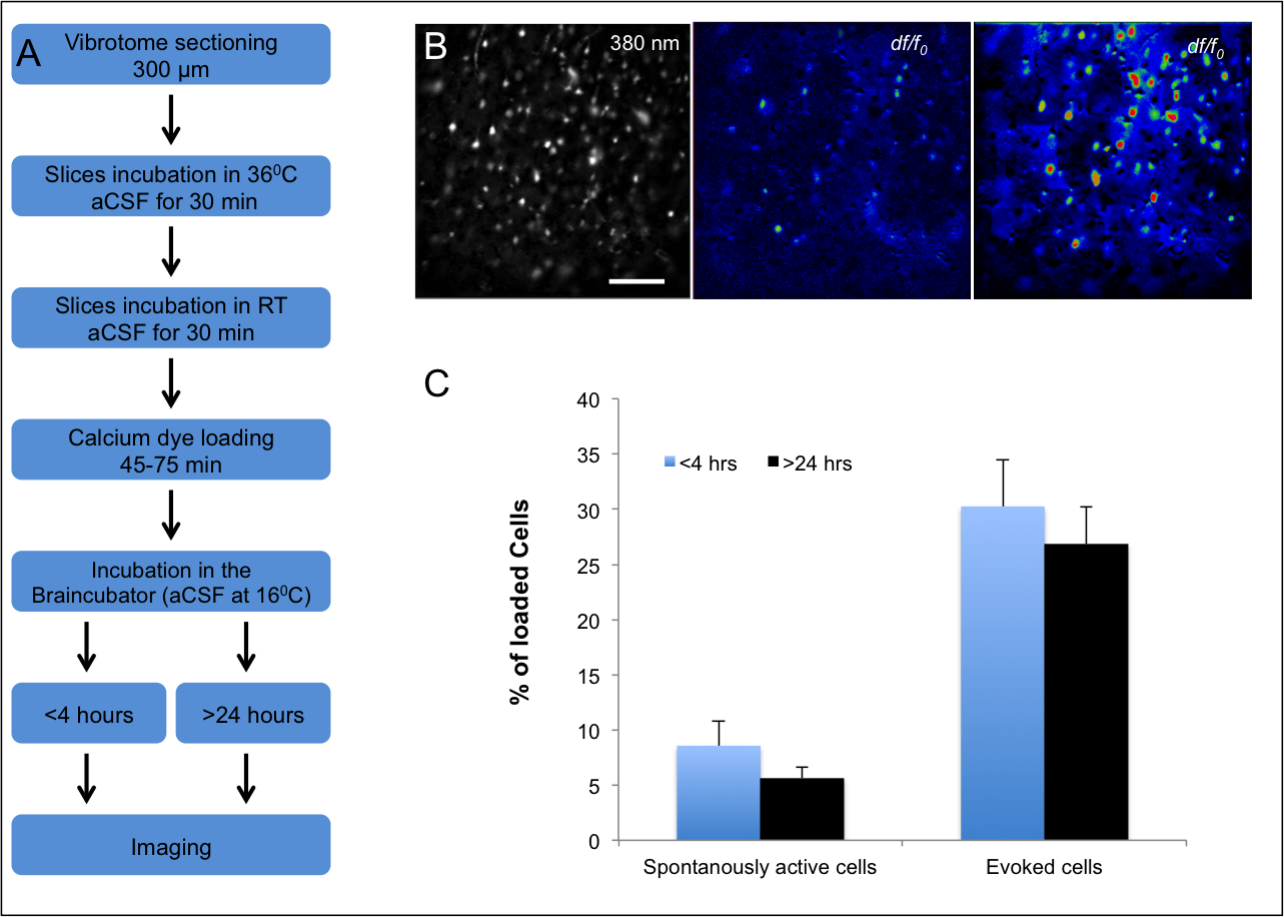
**Calcium imaging in acute brain slices** A) Flow diagram illustrating the slicing and calcium AM dye loading protocol in brain slices. B) Fluorescence images of a neocortical slice shows ubiquitous staining of cortical neurons and glia with Fura-2-AM (left image; 380nm excitation; 20x objective; scale bar 100 μm). Application of 30 mM KCl, caused a noticeable increase in 340/380nm ratio relative to F_0_ (*dF/F*), depicting three classes of cells according to their staining pattern. Left–Loaded cells; Middle–Spontaneously active cells (imaged as ratiometric changes before the application of KCl) and Right–Evoked cells (following 30mM KCl). Red and blue correspond to high and low Ca^2+^ concentrations, respectively C) Bar graph depicting the percentage of spontaneous and evoked cells, out of the total loaded cells within the field of view indicating a slight yet insignificant decrease in the both spontaneous and evoked cells following >24 hours in the Braincubator (p>0.9; two tailed student t-test).

This study aimed to demonstrate the length of time acute brain slices and excised retinae, loaded with calcium indicators, can be maintained to produce reliable and reproducible data about cell function. To increase neuronal tissue viability for more than 24 hours, we incubated the slices in the Braincubator, which provides a highly controlled environment, including pH, temperature, carbogen flow and low bacteria levels as reported previously [16,17]. To characterize the functional activity of neurons and glia by their calcium signals, we have used ratiometric (Fura-2 AM) and non-ratiometric (Fluo-4 AM) dyes that are widely employed to measure intracellular calcium signals. In all experiments, the tissue was loaded with the AM dye 1–2 hours after slicing, incubated for 45–75 min at either room temperature (Fluo-4 AM) or 37°C (Fura-2 AM), and transferred back into the Braincubator for incubation until experimental use (Fig 1).

**Fig 2:**
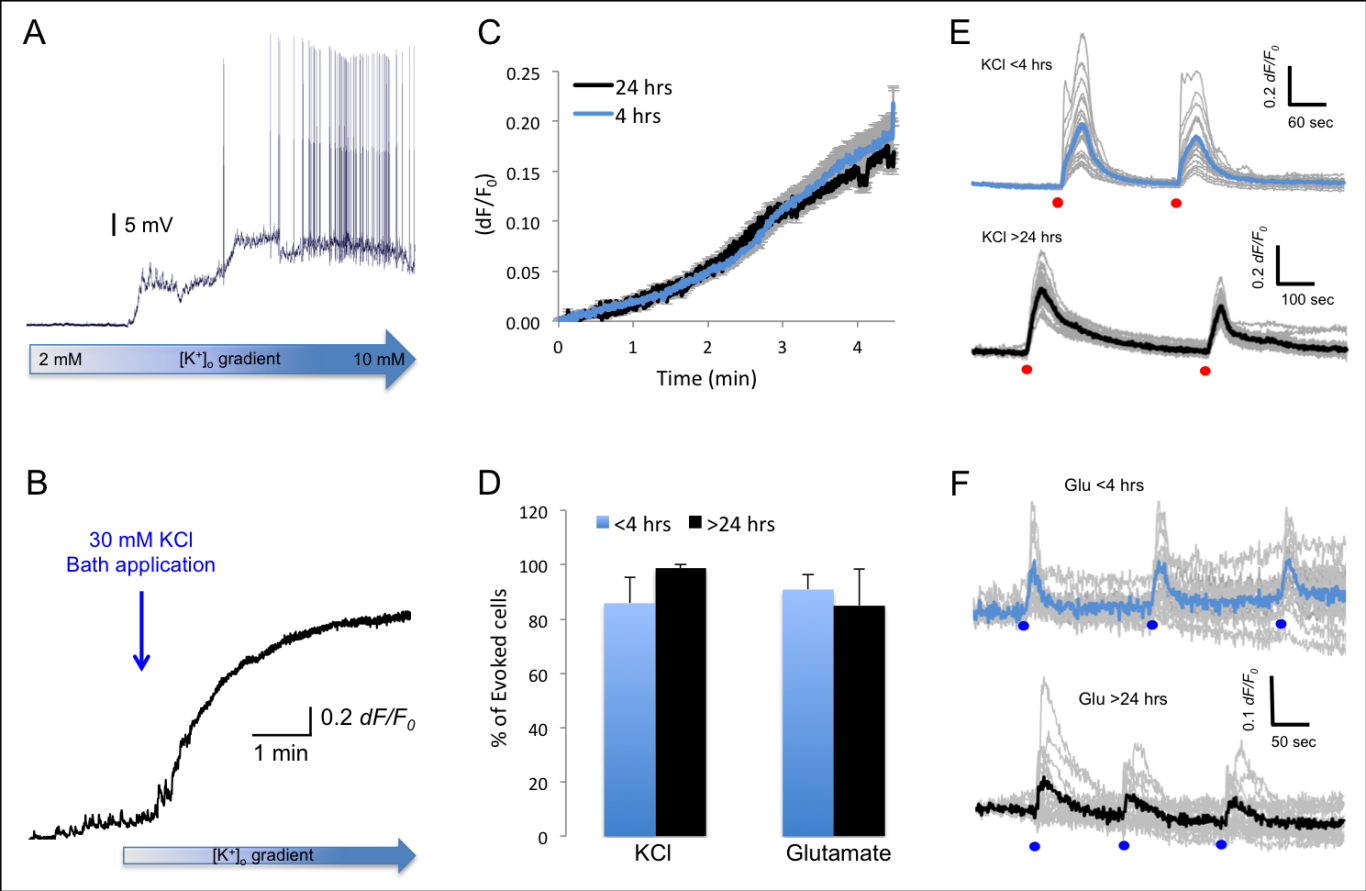
**The impact of depolarization on calcium signals** A) Bath application of KCl (10 mM) caused depolarization of the membrane potential of a layer 5 pyramidal neuron (somatosensory cortex), measured by whole-cell patch-clamp. B) Representative trace of a single cortical neuron shows spontaneous calcium transients followed by a large increase in dF/F following bath application of KCl (blue arrow). C) Plots of the average increase in calcium concentration in evoked cells from slices that were imaged <4 hrs (blue; n = 190 cells) and >24 hours (black, n = 236 cells) post slicing show similar kinetics. Measurements were aligned to the onset of KCl application. D) Bar graph depicting the percentage of evoked cells that recover after short local application of KCl (30 mM, 1 Sec; n = 4) or Glutamate (100 uM, 1 Sec; n = 4), in slices that were incubated for <4 hrs and >24 hrs. E) Intracellular calcium signals following repetitive short term application of KCl (30 mM; 1 sec). Grey–calcium traces in single cells; Blue–Average trace in a slice recorded <4 hrs post slicing; Black trace–average calcium signal recorded in a slice >24 hrs post slicing. Red dots indicate the time points of local KCl application. F) Intracellular calcium signals following repetitive local application of Glutamate (100uM, 1 Sec). Grey–calcium traces in single cells; Blue–average calcium trace in a slice recorded <4 hrs post slicing; Black trace–average calcium signal recorded in a slice >24 hrs post slicing. Blue dots indicate the time points of Glutamate application.

In all experiments, the cell bodies of the neurons were monitored individually and simultaneously for their calcium responses. Consistent with previous studies [27,28], we have divided the cells within the field of view into three classes according to their calcium signalling pattern. The first group contained cells that showed a basal level of staining indicating they were loaded with the dye and their membrane integrity was maintained (Loaded Cells–LC; Fig 1A). The second group contained all the cells that showed a spontaneous increase in intracellular calcium concentration, likely following transmitter release or opening of the internal calcium stores. These calcium transients were brief with various durations and intensities, which occurred in a repeatable manner without any external stimuli (Spontaneously active cells; Fig 1B). The third group contained cells that showed a large, prolonged and synchronised increase in calcium concentration following application of an external stimulus (30 mM KCl or 100 μM Glutamate), which led to a significant depolarization of the resting membrane potential (Fig 2A, S3 and S4 Videos) that resulted in activation of voltage gated channels and calcium influx into the cell (Evoked Cells; Figs 1C and 2).

In brain slices, the average number of cells that were still loaded with the AM dye (Loaded Cells) 24 hrs post loading was comparable to the number of loaded cells in slices that were imaged <4 hrs post loading (67±9 n = 18 and 66±7 n = 18 respectively; p>0.9). This indicates that long term exposure of the intracellular milieu to either fura-2 AM or Fluo-4 AM did not impact the membrane or dye integrity.

**Fig 3:**
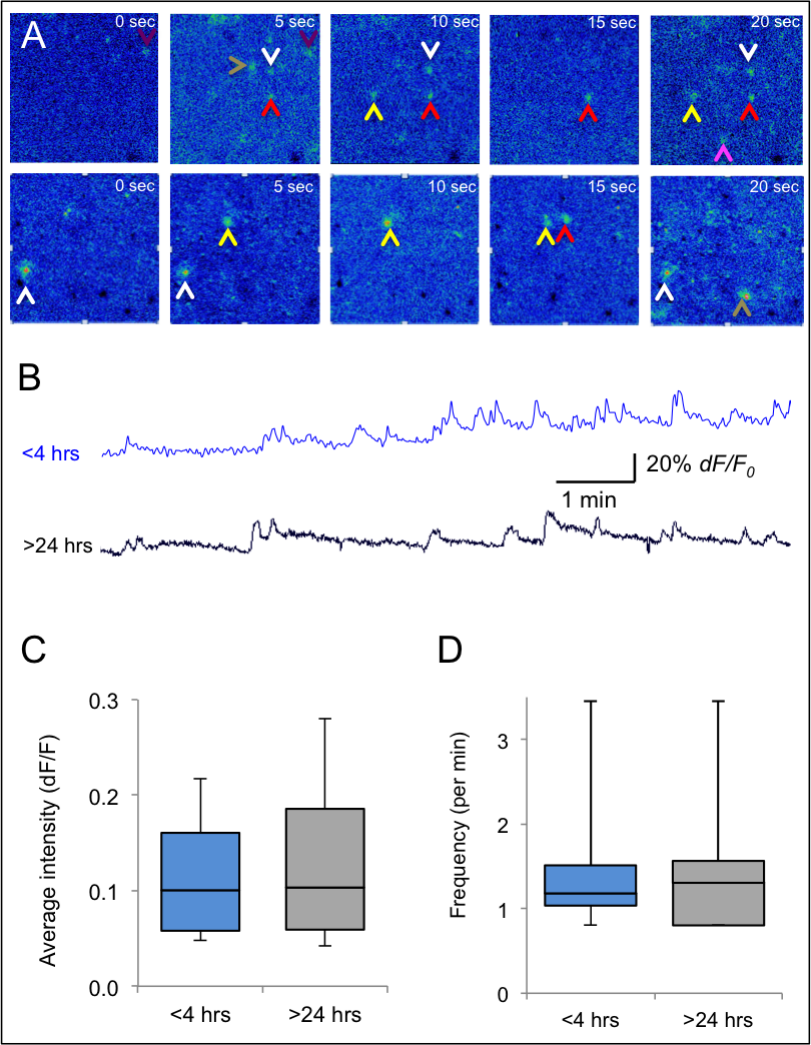
**Spontaneous Calcium signals in brain slices** A) Time lapse fluorescence microscopy images of slices loaded with Fluo-4 depicting spontaneous calcium transients. Top–Images from slice <4hrs post slicing; Bottom—Images from slice >24hrs post slicing. Arrows point to individual cells that show calcium transients (color coded). B) Sample traces of intracellular spontaneous calcium signals in cortical slices at the indicated times post slicing. C,D) Box plots of the average fluorescent intensity (C) and frequency (D) of the spontaneous calcium transients were not different at <4hrs and >24hrs post-slicing.

The average percentage of spontaneously active cells in the field of view was not significantly different between slices incubated for <4 hrs (8.5±2.2, n = 18 slices; Fig 1C) and those incubated for >24 hours (5.7±3.5, n = 18 slices; p>0.4). These results show that the majority of cells in slices that were incubated in the Braincubator for more than 24 hrs are still active and demonstrate normal calcium signals (Fig 2). Moreover, the average frequency of spontaneous calcium events imaged >24 hours post slicing was 1.39±0.18 events per minute (range 0.8–3.4 events per minute; Fig 3A) and was similar to the average spontaneous frequency in slices imaged <4 hours post slicing (1.38±0.16 events per minute, range 0.8 to 3.45 events per minute; n = 18; p>0.9, two tailed student t-test).

In spontaneously active cells, the average calcium increase was comparable between cells that were imaged for <4 hours post slicing and cells that were imaged >24 hours post slicing, indicating that the average calcium increase (0.05±0.01 vs 0.07±0.01 respectively, measured as changes in *dF/F*), median and dynamic range of the calcium signals did not change significantly (p>0.3, Fig 3).

To study calcium dynamics following the activation of voltage gated calcium channels, we added a high concentration of KCl to the recording bath [29]. As the resting membrane potential of neurons is extremely dependent on the extracellular K^+^ concentration [30], bath application of 30 mM KCl led to a slow depolarisation of the neuronal membrane potential (Fig 2), which led to an increase in calcium concentration, due to the activation of voltage operated calcium channels [29], NMDA receptors and release from internal stores (reviewed by [4]). On average, in ***acute brain slices***, the percentage of cells that showed an increase in intracellular calcium levels after the application of 30 mM KCl (Evoked cells) was comparable between the two groups of slices (27±3% in slices that were imaged <4 hours after slicing (n = 18) and 24±3% in slices that were imaged >24 hours post slicing (Fig 1; p>0.4 two tailed student t-test)). Moreover, in slices that were imaged <4 hours post slicing, the average increase in fluorescence following KCl application was similar to slices that were imaged following prolonged incubation periods (0.15±0.03 vs 0.19±0.03 respectively (*dF/F*); p>0.5, two-tailed student t-test).

As elevated cytoplasmic calcium levels are toxic and can lead to cell death [31], under normal physiological activity, elevated calcium concentrations are removed from the cytoplasm into internal stores by pumps and exchangers (off mechanisms; reviewed by [4]). Hence, restoration of calcium levels to baseline after stimulation signifies cellular viability. We therefore measured the percentage of **evoked cells** that exhibit recovery following short local application of KCl (1 Sec; 30 mM) or glutamate (1 Sec; 100 μM; Fig 2D–2F, S3 and S4 Videos). Our results indicate that under both conditions the percentage of evoked cells that restored calcium levels was comparable between slices that were measured <4 hrs post slicing (85±9% KCl; 90±5% Glutamate, Fig 2E) and slices that were incubated for >24 hrs in the Braincubator (98±1% KCl; 85±13% Glutamate; p>0.7; Fig 2F), indicating the second messenger system is functioning.

### Calcium signals in the retina

**Fig 4:**
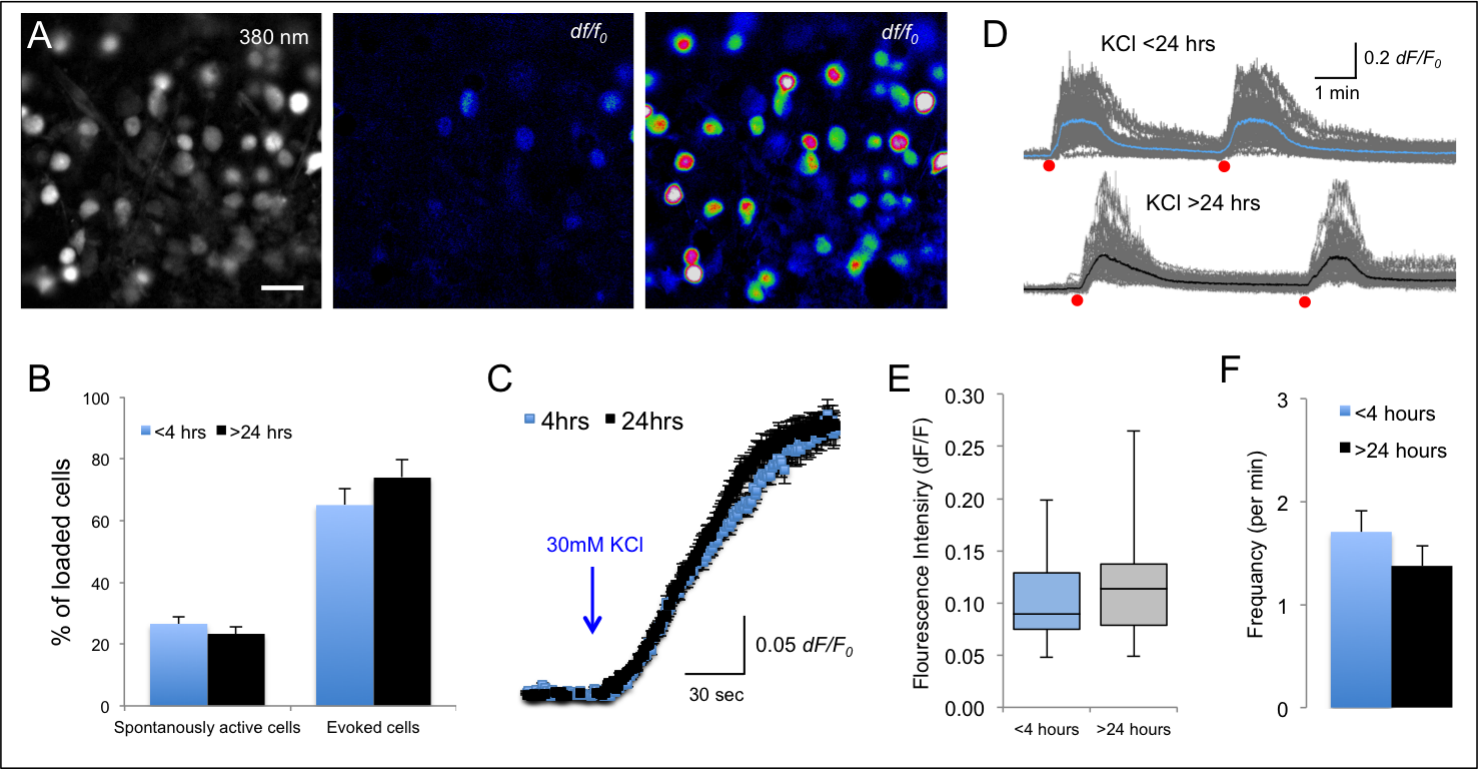
**Calcium dynamics in retinal slices** A) Fluorescence image of the ganglion cell layer of a wholemount retina shows ubiquitous staining with Fura-2-AM (left image; 380nm excitation; 60x objective; scale bar 30 μm). Application of 30 mM KCl, caused a noticeable increase in 340/380nm ratio relative to F_0_ (dF/F). Middle–Spontaneously active cells; right–Evoked cells (30mM KCl). B) Quantification of percentage of cells that were spontaneously active or responded to 30mM KCl with an increase in dF/F (evoked) were not different between 4hrs and 24hrs(p>0.4; two tailed student t-test). C) Plots of the average increase in calcium concentration in evoked cells from slices that were imaged <4 hrs (blue; n = 118 cells) and >24 hours (black, n = 180 cells) post slicing show similar kinetics and are not statistically different (P>0.4; two tailed student t-test). Measurements were aligned to the onset of KCl application. D) Intracellular calcium signals following repetitive short term application of KCl (30 mM; 1 sec). Grey–calcium traces in single cells; Blue–average trace in a retina recorded <4 hrs post slicing; Black trace–average calcium signal recorded in >24 hrs post slicing. Red dots indicate the time points of KCl application. E) Box plot describing the median and range of the average fluorescent intensity of the spontaneous calcium signals. F) Bar graph depicting the average frequency (per min) of spontaneous calcium signals imaged following <4hrs and >24hrs post-slicing (p>0.7; two tailed student t-test).

For many years, retinal wholemount has been used to study the function of retinal circuits as well as their synaptic activity [32, 33, 34]. However, the presence of the inner limiting membrane (ILM; formed by Müller cell endfeet), prevents direct access to the cells and therefore presents an additional challenge for dye loading and intracellular recordings. We therefore used papain proteolytic digestion to remove ILM prior to loading [23], which allowed ubiquitous loading of all cells in the retina, and particularly those in the ganglion cell layer (Fig 4A, S1 and S2 Videos). Retinae were then maintained by prolonged incubation time (>24 hours) in the Braincubator. As the retina is an exquisite light detector and responds strongly to the light used to excite the fluorescent indicator, we have used tissue from retinally degenerate *rd/rd* mice, in which rod and cone photoreceptors degenerate by the age of 4 weeks [34].

In wholemount retinae, the average percentage of both evoked as well as spontaneously active cells was comparable between tissue that was imaged >24 hours post slicing and tissue that was imaged <4 hours post slicing (Spontaneously active cells: 23±3% n = 8 vs. 27±3%, n = 9; p>0.4, two-tailed student t-test; Evoked cells: 74±5% vs. 65±9% respectively, n = 4, p>0.4 two-tailed student t-test; Fig 4B). Furthermore, the percentage of **evoked cells** that restored calcium levels to baseline following short local application of KCl (1 Sec; 30 mM) was comparable between retinae that were measured <4 hrs post slicing (94±3%) and retinae that were incubated for >24 hrs in the Braincubator (95±1%; p>0.7; Fig 4D), indicating on a functional second messenger system.

The average frequency of spontaneous calcium events imaged <4 hours post slicing was 1.71±0.2 events per minute (range 0.4–4.1 events per minute; Fig 4E) and was similar to the average spontaneous frequency in tissue imaged >24 hours post slicing (1.37±0.2 events per minute, range 0.37 to 4.1 events per minute; p>0.4, two tailed student t-test). Moreover, the average fluorescence increase in individual ***spontaneously active*** cells that were imaged <4 hours post slicing was comparable to the increase in individual cells that were imaged >24 hours post slicing (0.08±0.01 vs 0.08±0.01 respectively (*dF/F*); p>0.7), indicating that the dynamic range of the spontaneous calcium signal was not altered following prolonged incubation with the AM dye. Similarly, following KCl application, a fluorescence increase of 0.28±0.01 (*dF/F*; n = 118, Fig 4C) was measured in individual cells <4hrs of dissection, and was not changed significantly following prolonged incubation periods (0.26±0.01 (*dF/F)*; n = 180, >24 hours; p>0.5, n = 2 two-tailed student t-test). These results indicate that calcium dynamics remained intact for a minimum period of 24 hours post slicing.

## Discussion

Calcium signals, carried by alterations in intracellular calcium concentration, are an essential element in many neurophysiological processes, such as signal transduction, gene expression and neural plasticity [15], which makes them a primary indicator for the vital physiological functions of neurons. Intracellular calcium levels are controlled by ***on*** and ***off*** mechanisms that elevate and restore the calcium concentration to baseline respectively [4]. The ***on*** mechanisms involve initial calcium increase through voltage-operated channels (VOC’s), that act together with inositol- 1,4,5-trisphosphate receptor (IP_3_) to induce further release of Ca^2+^ from the internal stores. The ***off*** mechanisms are activated at high cytoplasmic calcium levels and involve activation of the second messengers system, specifically pumps and exchangers (e.g. Na^+^/Ca^2+^ exchanger, sarco-endoplasmic reticulum Ca^2+^ ATPase (SERCA)) to remove calcium ions out of the cytoplasm [4].

Calcium concentrations are strongly associated with neuronal electrochemical properties, which impact the calcium influx through transmitter gated (e.g., NMDA, nicotinic and purinergic receptors) and voltage dependent calcium channels [5], suggesting that calcium signals are the fingerprint of sodium spiking and network UP states, in which high calcium levels are maintained in precisely organized neuronal ensembles [35]. For this reason, the use of calcium AM ester indicators, such as Fura-2 AM and Fluo-4 AM, provides a robust method of imaging the activity in large populations of neurons simultaneously and therefore has become a central tool for the study of information processing in neurons and glia in the CNS. This information, which essentially is an integrated signal of sensations and behaviour encoded as patterns of neuronal network activity, results in different calcium concentrations and is further translated by proteins such as phospholipase C and calmodulin-dependent Kinase II (CaMKII), that activate and deactivate target proteins and thus lead to downstream processes [4,7]. In many cases, differences in the calcium concentration might lead to opposing cascades, as happens during synaptic plasticity, in which low increase leads to LTD, while high increase in calcium levels result in LTP.

The proposed study had two aims: the first was to investigate whether the effectiveness of calcium AM staining persists following long incubation periods post cell loading, and the second aim was to test whether the functional properties of calcium signals in acute brain slices and wholemount retinae altered following extended incubation periods in a tightly controlled environment.

Our results indicate that under standard aCSF incubation in the Braincubator at 16°C, both Fura-2 AM and Fluo-4 AM remained within the cells long after loading (>24 hours; Figs 1 and 4). Furthermore, analysis of the calcium signals within individual cells in slices that was imaged >24 hrs after loading, showed that the number of loaded cells, spontaneously active cells and evoked cells was similar to slices that were imaged 2–4 hours after loading (Figs 1 and 4). Moreover, the basal intracellular calcium concentration in cortical cells following >24 hours post slicing was 43±3 nM, and 50±3 nM for retinal cells, which is within the normal range, as previously reported by [4]. Additionally, the percentage of evoked cells that recovered following short local application of either Glutamate or KCl was comparable between slices that were measured <4hrs and >24hrs post slicing (Figs 2 and 4). In all, these results indicate that long exposure of the AM dyes to the intracellular cytoplasm did not alter the intracellular calcium concentration or viability of the neurons. Moreover, the increase in calcium concentrations after >24hrs (either during spontaneous or evoked activity) was comparable to calcium increases in fresh slices (Figs 2–4), demonstrating that the functional range of the dye remained effective and calcium signals were not altered following long incubation periods. Taken together, these data suggest that experiments using calcium-imaging on neuronal tissue can be reliably completed for at least 24 hours after dissection/slicing and dye loading, when the tissue is incubated in the Braincubator.

While acute brain slices offer several advantages (i.e. extracellular ionic control, mechanical stability, preserved anatomical structure and mostly preserved local network communication), cell culture techniques exhibit anatomical reorganisation and altered expression profile of ion channels and receptors, which develop over time (reviewed by [36]). Hence, extending the viability of acute brain slices may result in alterations of the expression pattern of ion channels and receptors, as occurs in cultured slices.

Retinal wholemount present an additional challenge for calcium imaging, as the endfeet of Müller cells form a barrier that does not allow the penetration of the dye into the cells. In this paper, we have used a proteolytic enzyme, papain, that digest the membrane endfeet and exposes the cells [23]. This procedure is been highly efficient in removal of the outer membrane layer (ILM) and allows ubiquitous dye loading. Although it was acknowledged by Velte and Masland (1999) that some retinal ganglion cells may be damaged by the papain treatment, our results demonstrate activity in ~75% of stained cells, suggesting that the majority of neurons of the retinal ganglion cell layer are intact and functional. It remains to be seen, however, how network activity of these cells is affected by the removal of the Müller cell endfeet. Considering the length of this procedure, incubation in the Braincubator provides major benefits, as viability as well as physiological properties measured through calcium transients remained intact >24 hours post slicing.

One of the advantages of calcium imaging over electrophysiological recording is the spatial resolution. Indeed, voltage sensitive dyes can also support high spatial resolution, however, voltage sensitive dyes are highly toxic to the cells and therefore cannot be incubated for long periods. In this paper we used calcium imaging to show that using highly controlled environment incubation system extends the viability of acute brain slices and retinal wholemount. Moreover, intracellular calcium transients were indistinguishable >24 hours post dissection to freshly prepared tissue, indicating their functional properties are still intact. Furthermore, the long exposure of the AM dye did not alter calcium signals, showing that it was not toxic and still efficient to use. We believe that this method can be applied to many more AM dyes, including selective markers to NO (DAF-2DA; see [37,38]). Beyond the benefits of extending slice viability and calcium imaging as discussed above, the impact on animal welfare is potentially very important. Extending the recording time available per slice increases the yield per animal, providing the possibility of significantly reducing the number of animal used in experiments of the type described in this paper.

## Supporting Information

S1 Video Calcium imaging of retina wholemount <4 hours post slicing. (MP4)

S2 Video Calcium imaging of retina wholemount >24 hours post slicing. (MP4)

S3 Video Calcium imaging of acute brain slices >24 hours post slicing with local KCl application. (MP4)

S4 Video Calcium imaging of acute brain slices >24 hours post slicing with local Glu application. (MP4)
